# An eye tracking study: positive emotional interface design facilitates learning outcomes in multimedia learning?

**DOI:** 10.1186/s41239-021-00274-x

**Published:** 2021-07-20

**Authors:** Xian Peng, Qinmei Xu, Yufan Chen, Chenying Zhou, Yuqing Ge, Na Li

**Affiliations:** 1 National Engineering Laboratory for Educational Big Data, Central China Normal University, Wuhan, 430079 Hubei China; 2 Learning and Cognitive Science Laboratory, College of Education, Zhejiang University, Hangzhou, 310028 Zhejiang China

**Keywords:** Interface design, Positive emotion, Multimedia learning, Learning outcomes

## Abstract

Unlike the other studies on emotional design in multimedia learning, the present study differentiated the two confounding variables of visual interface design and structured content to manipulate the instructional material. Specifically, we investigated how the visual aesthetics of positive emotional interface design influenced learners’ cognitive processes, emotional valences, learning outcomes, and subjective experience. Eighty-one college students took part in the experimental study. They were divided into the three experimental groups: a holistic layout of positive emotional design group (HPED), a local layout of positive emotional design group (LPED), and a neutral emotional design group (ND). By using a mixed approach of questionnaires and eye tracking, we further explored the differences among the three groups in cognitive processing, learning outcomes, and subjective experience. Results indicated that the LPED group invested higher cognitive effort, put more attentional focus in the relevant knowledge content module, and achieved better learning performance (i.e., retention and transfer tests) in contrast to the HPED group and the ND group. However, no significant difference in dynamic changes of emotional state among the three groups was detected. The analytical results can provide researchers and practitioners with valuable insights into the positive emotional design of multimedia learning, which allows for the facilitation of mental engagement, learning outcomes and subjective perception.

## Introduction

Multimedia learning has become a mainstream means of formal and informal settings, such as, institutions of higher education, full-time organizations, and enterprises (Li et al., 2020). The design of multimedia instruction and the adjustment of learning materials bring richer resources and presentation forms to teaching and learning, which will directly affect learners’ cognitive processes and academic performance (Mayer, 2019; Mutlu-Bayraktar et al., 2019). Emotional design combined with the use of different emotional elements during learning processes is an essential part of multimedia instruction environment. Analysis of emotional design of multimedia materials has been shown to be effective in understanding and revealing the underlying mechanism of multimedia learning (Mayer & Estrella, 2014; Münchow & Bannert, 2019; Park et al., 2015b; Plass et al., 2014; Um et al., 2012). Compared with external emotional induction (i.e., external induction of stimuli through some procedures such as watching funny or annoying videos before a learning task) (Isen et al., 1987; Liew & Tan, 2016), internal emotional design (i.e., internal induction of emotions through the design of materials during the process of multimedia learning) is more stable to evoke learners’ emotions and easier to be administrated in the actual teaching practices (Mayer & Estrella, 2014; Park et al., 2015a; Stark et al., 2018). However, the question how to apply the internal emotional principles in designing the optimized multimedia learning materials is still not well answered.

Several studies on multimedia learning have indicated that the aesthetically appealing design of learning materials (e.g., visual features, design layout, color and sound embedded in the multimedia environments) can induce positive perception and intrinsic motivation (Heidig et al., 2015; Moshagen & Thielsch, 2010; Tractinsky et al., 2000; Wolfson & Case, 2000). Subsequently, these affective measurements have a direct impact on learning outcomes and cognitive investment. Moreover, warm colors and anthropomorphic shapes considered two crucial design aspects that induce positive emotion state are found to be effective in the multimedia instruction. The multimedia design with emotional elements can increase or decrease learners’ mental engagement to regulate their cognitive processes and learning outcomes (Beege et al., 2018; Ng & Chiu, 2017; Plass et al., 2014; Stárková et al., 2019; Um et al., 2012). For example, Um et al. (2012) examined the influence of positive emotions evoked before learning or during learning on learners’ cognitive and affective processes. The results indicated that the emotional design principles with warm colors and round shapes in a multimedia learning environment could induce positive emotional state and motivate them to achieve better learning gains. Therefore, this study is also expected to consider the warm color and baby-like shape features integrated in the design of multimedia instruction during learning, in order to evoke positive subjective experiences and facilitate learning effectively. In addition, the eye tracking indicators, such as first fixation time, total fixation time, and total fixation count, enable us to grasp a detailed overview of the measurement of mental representation of online cognitive process, for the purposing of revealing the potential effects of emotional layout integration in learning with multimedia environment (Park et al., 2015a, b; Stark et al., 2018).

**Fig. 1:**
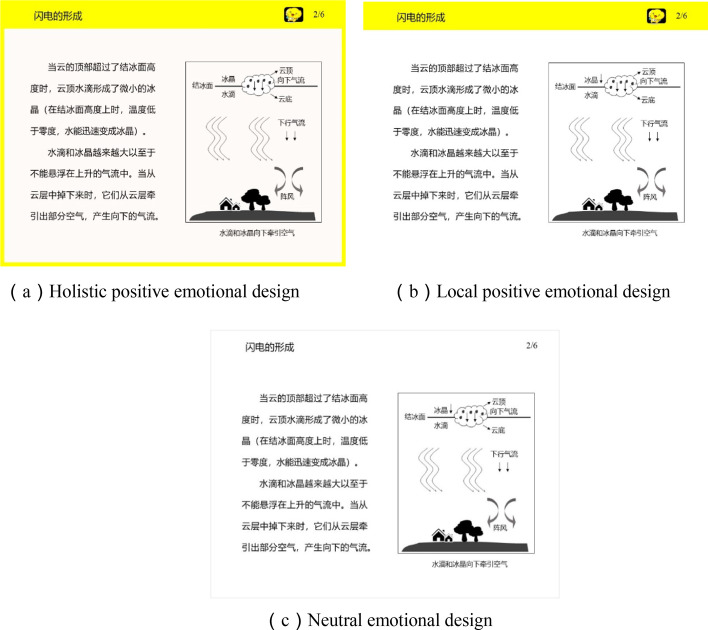
Screenshot of the learning material in the positive versions (up; HPED and LPED) and the neutral version (down; ND)

Taking into consideration that the emotional design of multimedia learning material (e.g., the reserve of aesthetic design knowledge, the redesign of instruction elements associated with textual content or graphics) greatly improves the instructors’ practical workload and operational difficulty, the question arises whether it is possible to simplify the procedures of material design itself and reduce the operational difficulty of teaching practitioners. To our knowledge, few studies focusing on the emotion design of aesthetic interface in multimedia instruction have been explored. Moreover, study by Heidig et al. (2015) suggested that objective system qualities such as structure, layout, or website aesthetic design could trigger emotional responses from users. To sum up, this study is to decompose the interface manipulation and content manipulation variables in designing multimedia material owing to possible confounds. We then propose a new design pattern of positive emotion interface layout of the learning program (holistic layout vs. local layout, see Fig. 1). It does not add the extra new content to the study material and is more consistent with the practical presentation form of multimedia courseware used by instructors. By means of the new positive interface design version of multimedia learning, our study aims to examine the effect of induced positive emotions with two kinds of emotional characteristics (i.e., warm colors and anthropomorphic shapes) during learning on learners’ cognitive processes, emotional states, learning outcomes, and subjective perception.

## Related work

### Emotional design of multimedia learning

According to the Cognitive Affective Theory of Learning with Media (CATLM) proposed by Moreno (2005, 2009), cognitive, motivational and affective factors should be jointly considered in constructing the coherent mental representations of multimedia information. The affective mediation hypothesis states that affective aspects have a moderating effect on learning outcomes by increasing or decreasing motivational and cognitive involvement (e.g., interest, motivation, meta-cognition). With regard to the function of positive emotions, two conflicting assumptions are proposed. Some studies supported the emotions as extraneous cognitive load assumption that the addition of any features designed to evoke positive emotions in teaching materials would increase external cognitive load and thus impaired learning (Harp & Mayer, 1997; Pekrun, 1992; Sweller, 1988, 2011). However, others addressed the emotions as facilitators of learning assumption that the positive emotions evoked during learning process had a direct positive influence on learning or mediated the motivational aspects to affect learning (Astleitner, 2000; Isen & Daubman, 1984; Plass et al., 2014; Stárková et al., 2019; Um et al., 2012). Emotional design refers to the use of different design elements in manipulating the process of multimedia instruction and is adopted to impact learners’ moods and facilitate their learning achievements (Mayer & Estrella, 2014). Furthermore, emotional design, on the basis of the sequence of time phases of the experimental operation, can be categorized into two types of instructional materials design for the purpose of inducing learners’ emotions: external emotional induction before learning and internal emotional design during learning.

A combination of the two positive emotional design approaches by Um et al. (2012) was adopted to investigate the question whether the experimental multimedia materials could be designed to induce positive emotions that would improve affective feelings and facilitate learning. Learners were provided with affective stimulation (positive emotion and neutral emotion) either by reading the emotional statements before learning or by redesigning the learning materials (background in orange and red colors, content objects dominated by warm colors, round, and face-like shapes) during learning. The results demonstrated that employing the principles of positive emotion design could invoke learners’ positive emotions and enhanced their comprehension and transfer performance. Regarding the research of external positive emotion stimulation before learning, Knörzer et al. (2016a, b) examined the impact of experimentally induced emotions (positive, neutral, negative) on multimedia learning by the use of the playing of music pieces and the recall of happy or sad situation stories. The results indicated that an induced negative emotional state had a facilitating effect on learning outcomes, whereas a suppressing effect of an induced positive emotional state on learning processes was observed. Regarding the study of internal positive emotion design during learning, Uzun and Yıldırım (2018) classified four emotional groups (the colorful design group, the anthropomorphic design group, the anthropomorphic design and sound effects group, and the neutral design group) to compare the effects of different emotional design patterns on affective assessment measures, cognitive processes, and learning outcomes. The results found that with the increase of the number of emotional design features, learners’ positive emotions were typically strengthened. The color design group invested more mental effort than the neutral design group, whereas the anthropomorphic design and sound effects group invested less mental effort than the color design group. Additionally, the color design group performed better on the recall test than the control group. A study by Heidig et al. (2015) adopted the concepts of visual aesthetics of websites (i.e., usability, classical and expressive aesthetics) to differentiate the impact of relevant positive and negative design features in the emotional design of multimedia learning material, excluding from the external emotion induction. Unexpectedly, the design differences in aesthetics or usability had no direct effect on learners’ emotional states and test performances, whereas learners’ emotions, to a large extent, influenced learners’ intrinsic motivation.

In reference to the studies reported above, the present study retained the color and anthropomorphic shape of two crucial independent variables that had been shown to be effective in affecting learning processes and outcomes (Park et al., 2015a, b; Plass et al., 2014; Stárková et al., 2019; Um et al., 2012; Uzun & Yıldırım, 2018).

### Eye tracking technology in multimedia learning

Eye tracking technology, considered an effective and advanced methodology in multimedia learning, is capable of recording the visual processing of information acquisition and revealing the rules of cognitive activity, instead of behavioral evaluation, questionnaires, and self-reporting measurements (Rodrigues & Rosa, 2019). As Mayer said (2019), objective measurements were required to directly capture and explain learners’ cognitive engagement in the process of multimedia learning. According to the basic assumption of cognitive processing (CATLM) (Moreno, 2005, 2009), cognitive activities are categorized into three primary steps: selecting, organizing, and integrating. Select process is regarded as the primary premise of perceiving and maintaining relevant information while ignoring the recognition of irrelevant information; organization process is seen as the retrieve and understanding of the selected information in working memory; integration process is considered as the visual transitions of various items (e.g., textual and graphic information) presented from learning material (Schüler, 2017), in order to construct a consistent mental representation model. Eye movement measurements enable researchers and practitioners to observe learners’ temporal fluctuations (e.g., what specific items they look at in the scene, and how they fixate in the change of visual attention) and make references about these cognitive processes (Hyönä, 2010).

Few studies have been conducted to investigate the influence of emotional design with various features on eye viewing behaviors as well as the association between the eye movement measurements and learning outcomes during the process of multimedia learning, which may be attributed to the operation difficulty and high cost of eye tracking devices (Park et al., 2015a, b; Rodrigues & Rosa, 2019; Stark et al., 2018). Nummenmaa et al. (2006) indicated that emotional graphics could attract the participators’ attention and increase their level of visual attention, thereby extending the fixation time on emotional graphics. A study by Park et al. (2015a, b) employed eye movement technology to capture learners’ emotional states and underlying cognitive processes during multimedia learning. The findings showed that while the anthropomorphic emotional design did not cause learners’ positive emotions in the learning environment, the eye movement data demonstrated that the anthropomorphic emotional design did attract learners’ focuses towards the specific appealing objects. Moreover, learners in a positive emotional state with the anthropomorphisms design performed the highest learning score. Generally, this study provides part evidences to interpret the relationship between information processing derived from eye movement measurements, emotion, and learning outcomes in multimedia learning. Stárková et al. (2019) shown that learners with the anthropomorphized pictorial elements in multimedia learning materials allocated significantly more visual attention during initial observation but not overall. Unexpectedly, anthropomorphisms had no significant impact on immediate and delayed learning.

Except for using eye tracking technology in multimedia learning researches, Le et al. (2018) adopted the physiological indicators of heart rate variability to measure the effect of emotional design principles on learners’ mental engagement. The results found that participants in the positive emotion group had a better performance on subsequent retention and showed a greater decline in the high-frequency band of heart rate changes than those in the neutral design group. As the inner psychological characteristics of people were difficult to be definitely explained through subjective personal reports, this study added the instrument of eye tracking measurement to reveal the potential factors and relationships of influencing learning processes and learning outcomes behind the reported data.

## Research questions and hypotheses

To induce positive emotion during learning and improve the generalizability of teaching practices, this study uses the manipulations of internal emotion design (e.g., the combination of warm and anthropomorphic elements) by separating the confounding factors of interface design layout and content design layout in the multimedia learning environment. Specifically, in line with the theoretical assumption of positive emotion design, the goal of this study aims at investigating the effect of interface layout design (with holistic layout, with local layout, and without interface layout, see Fig. 1) incorporating positive elements on learners’ emotional states, learning outcomes, visual attention processes by eye tracking, as well as learners’ subjective experience, that is, invested cognitive mental load, perceived task difficulty, intrinsic motivation, and perceived task achievement. Notably, we adopt the version of static presentation of multimedia instruction, rather than the dynamic animation, according to the design principles of previous researches (Désiron et al., 2018; Mayer, 2005; Mayer & Estrella, 2014; Stark et al., 2018). The contiguity condition of text and pictures are close in time or space, and the verbal and pictorial information are highly correlated. Generally, this study addresses the following four questions:

1. *Does the interface layout of positive emotion design, to what extent, facilitate learners’ learning outcomes? (Hypothesis 1)*

It is assumed that the interface layout of positive emotion design will lead to better learning outcomes (i.e., recall test, comprehension and transfer tests) compared with the original multimedia design.

2. *Does the interface layout of positive emotion design combined with multimedia learning, to what extent, induce learners’ emotions? (Hypothesis 2)*

Based on the extension of emotional design assumptions with regard to the positive emotion design in multimedia learning, it is postulated that both (holistic and local) layout of positive emotion designs lead to higher learners’ positive activation compared with the original picture and text design (neutral group without any emotional features embedded in the multimedia material).

3. *Does the interface layout of positive emotion design, to what extent, change learners’ visual attention processes derived from eye tracking measurements? (Hypothesis 3)*

In line with the emotional design assumption “making basic features visually appealing can activate and guide cognitive processes during learning” (Mayer & Estrella, 2014; Um et al., 2012), it is posited that the interface layout of positive emotion deign is attractive for learners to invest longer fixation duration and more fixation counts on relevant content parts of learning program. Further, it is assumed to lead to longer fixation duration and more fixation counts focused on text elements rather than graphic elements during multimedia learning. Specifically, based on the principle of overall priority in perceptual processing, the holistic layout is assumed to attract more attention than the other groups.

4. *Does the interface layout of positive emotion design, to what extent, impact learners’ subjective experiences, that is, invested cognitive mental load, perceived task difficulty, intrinsic motivation, perceived task achievement? (Hypothesis 4)*

In combination with positive emotional features including warm colors and anthropomorphic shapes in multimedia instruction, it is expected to find that learner in the groups with the interface layout of positive emotion design will present higher ratings of invested cognitive mental load, intrinsic motivation, perceived task achievement and lower ratings of perceived task difficulty, compared with the original multimedia design.

## Method

### Participants and experimental design

This study recruited 83 college students (48 female; age: *M* = 22.48, *SD* = 1.54) from Zhejiang University in China, who received 20 RMB for their participation. Their majors include education (16.87%), humanities (10.84%), psychology (9.64%), art (4.82%), economics and management (4.82%), engineering (12.05%), energy (9.64%), computer science (9.63%), chemistry (4.82%), biology (3.61%), agriculture (2.4%), mathematics (2.4%), medicine (2.4%), and others (6.02%). Due to the loss of eye movement data (the sampling rate was less than 70%), 80 college students’ eye tracking data were eventually retained. We conducted a one-factorial experimental between-subjects design with the interface design layout factor: a holistic layout of positive emotional design group (HPED) through the design of learning material, a local layout of positive emotional design group (LPED) through the design of learning material, and a neutral emotional design group (ND) with the original learning material. The students were randomly assigned to one of the three experimental groups, considering eye vision (e.g., glasses or contact lenses) in order to control eye tracking results.

### Learning material

The formation of lightning served as the experimental materials in the multimedia learning environment (Mayer & Moreno, 2003). The courseware content introduced the basic principle and main process of lightning for educational knowledge. The learning program consisted of 7 chapters with written texts and the corresponding static picture, which was displayed on a fixed eye tracker terminal device. The holistic layout of positive emotional design group (HPED) and the local layout of positive emotional design group (LPED) contained warm colors and anthropomorphic shapes (i.e., the yellow outer frame, the background color of the orange saturation, the anthropomorphic icon), whereas the neutral group only retained the original content itself (Fig. 1). The whole learning process lasted 7 min; each screen kept a paced learning time.

To maintain the integrity and consistence of the courseware information represented, the same amount of learning elements, the position of picture and text, the same multimedia learning design principles for the positive and the neutral instructional materials were adopted (Mayer, 2005).

### Measures

#### Learning outcomes

This study assessed learning outcomes from the three aspects: retention test, comprehension test, and transfer test. The retention test measured how well learners remembered what they have studied, including an essay question on how lightning works with 16 scoring points. The comprehension test was designed to test learners’ basic understanding of knowledge concepts. It consisted of 12 multiple choice items with 12 scoring points. The transfer test was used to test learners’ ability of knowledge transfer, which required learners to construct and process knowledge and completed the deep processing of meaning learning. It included 4 open questions about the formation of lightning, with 3 scoring items for each question, a total of 12 points. For example, “How can the intensity of lightning be reduced?”. Two research assistants rated and resolved the differences of scores by discussion and negotiation on the items, with an inter-rater reliability of *r* = 0.765.

#### Emotional state

The Positive Affect Scale (PAS) derived from the Positive and Negative Affect Schedule (PANAS; Watson et al., 1988) was utilized as an experimental manipulation in the present study. PAS contained 10 sub-items related to positive emotional feelings (i.e., interesting, exciting, strong, enthusiastic, proud, alert, inspiring, determined, concentrative, and active). The intensity of positive emotions they experienced on each sub-item was measured on a five-point Likert scale, ranging from 1 (very low) to 5 (very high). Each participants’ positive emotional score was equal to the sum of their responses to the 10 sub-items. Moreover, we used PAS to evaluate participants’ emotional values twice: the first time before learning (PA t1; *Cronbach's α* = .843) and the second time after formal learning (PA t2; *Cronbach's α* = .922).

#### Eye tracking

The experimental instrument was Tobii Pro Spectrum 600 from Sweden and its supporting software to detect learning eye movements, with the spatial sampling rate of 600 Hz. It was a fixed tabletop device using the corneal infrared tracking technology. The stimulus was presented on a 19-inch Dell Liquid crystal display with a resolution of 1280 × 1024 pixels. For analyzing the participants’ eye movements, the predefined *Areas Of Interest* (AOIs) involving the textual or pictorial information of the multimedia learning material was depicted. As the main indicators of cognitive activities, we adopted the entire fixation duration and fixation count on AOIs (i.e., instructional title, textual and pictorial content) to discriminate the participants’ information processing patterns.

#### Additional dependent measures

As a vital measurement index, cognitive load developed by Pass (1992) were used to assess the learners’ mental effort (“How much mental effort did you put into the preceding learning task?”) and perceived learning difficulty (“How difficult was it to complete the tasks?”) consisting of a nine-point Likert scale, ranging from 1 (very little) to 9 (very much). With regard to intrinsic motivation, we adopted a self-report questionnaire containing an 8-item test with seven-point Likert scale developed by Isen and Reeve (2005) (e.g., “The study task is enjoyable.”; ranging from 1 (strongly disagree) to 7 (strongly agree) (*Cronbach's α* = .934). Interface design (“How much did you like the interface layout of multimedia learning material?”), learning experience (“How satisfied were you with the previous learning material?”) and perceived learning task (“How well did you think you had completed the task?”) were measured on a seven-point Likert scale.

#### Control measures

We used the subjective self-rating scale to evaluate participants’ prior knowledge from the following two aspects (Mayer & Moreno, 2003). On the one hand, the subjective scale covered 5 multiple-choice items of familiarity with meteorological knowledge, for example, “I know what is a warm front?”. It adopted the five-point Likert scoring method, ranging from 1 (very unfamiliar) to 5 (very proficient) (*Cronbach's α* = .66). On the other hand, participants were asked to responded to a self-rating question on meteorology knowledge (“How much did you know about meteorology?”), with a five-point Likert scale (very little to very much). Thus, participants’ prior knowledge level was equal to the sum of the above two items’ scores. Additionally, we regarded the *background questionnaire* associated with sociodemographic information as control measure.

### Procedure

This experiment adopted the screen stimulation paradigm in a laboratory setting for the duration of 40 min, including the pre-test phase, the learning phase and the post-test phase. Before the beginning of the test, the investigator introduced the purpose and requirements of the experiment to the subjects. The subjects were required to fill in their basic personal information (gender, age, specialty, etc.), complete the consent form and the assessment of prior knowledge, and complete the first positive emotional self-rating scale (PA t1). In the formal stage of the experiment, the subjects first needed to perform 5-point automatic eye movement calibration. Then, after the calibration, they needed to browse and study the learning contents of each courseware page within the specified time. In the post-test phase of the experiment, the subjects were required to fill in the second positive emotional self-rating scale (PA t2), cognitive load scale, learning motivation scale, learning experience and perceived learning task. Moreover, they should answer questions about recognition test, comprehension test and transfer test through computer programs. Note that we followed ethical principles in conducting the study of human subjects. SPSS software was used for statistical analyses and p-value was set at .05.

## Results

### Preliminary analysis

One-factorial Analysis of variance (ANOVA) was administrated to measure the three different groups’ prior knowledge on meteorology as between-subject variable. The results showed that there was no statistically significant difference among the three groups for the control measure on prior knowledge, *F* < 1.

### Learning outcomes

To answer the research question 1, the results of retention, comprehension, and transfer scores for three groups are described in Table 1. One-factorial ANOVAs detected significant between-subjects differences with regard to retention score, *F*(2, 77) = 3.38, *p* = .039, η^2^ = .081, as well as transfer score, *F*(2, 77) = 8.33, *p* = .001, η^2^ = .178. Post hoc analysis indicated that the LPED group significantly outperformed the HPED group in retention score, LPED-HPED: Δ*M* = 2.28, *p* = .015; transfer score in the LPED group was significantly higher than those in the HPED and ND, LPED-HPED: Δ*M* = 2.28, *p* = .001, LPED-ND: Δ*M* = 2.28, *p* = .001. In line to the hypothesis 1, the interface design layout that incorporated some essential positive elements in multimedia instruction would lead to better learning outcomes, only for the mode of local interface layout of positive emotional design.

**Table 1:** Means and standard deviations of retention, comprehension and transfer scores for the three groups

	Group	M	SD	F	p
Retention score [max = 12]	HPED (n = 27)	7.41	3.66	3.38	.039
	LPED (n = 26)	9.69	3.32		
	ND (n = 27)	9.15	3.05		
Comprehension score [max = 16]	HPED (n = 27)	8.30	1.79	.83	.439
	LPED (n = 26)	8.81	1.17		
	ND (n = 27)	8.56	1.28		
Transfer score [max = 12]	HPED (n = 27)	3.85	1.10	8.33	.001
	LPED (n = 26)	5.12	1.58		
	ND (n = 27)	3.85	1.17		

### Emotional state

**Fig. 2:**
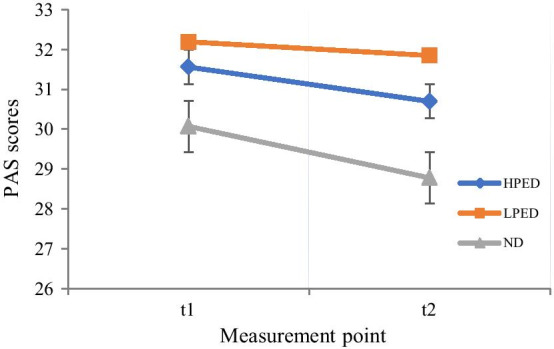
PAS values on the first and second measurement point for the three groups

To examine the changes of participants’ positive emotional state in multimedia instruction, we adopted a 2*3 RM-ANOVA with the measurement point of before and after learning as within-subject design and with the three experimental emotional interface design groups (HPED, LPED, ND) as between-subject design. The PAS values on the first and second measurement point for the three groups are depicted in Fig. 2. Unexpectedly, the results showed that there was no significant interaction effect between the measurement point and the three groups, *F*(2, 77) = .209, *p* = .812, *n.s*. Moreover, we did not find any main effects for the measurement point of the PAS and the positive emotional interface design group, *Fs* < 1, *n.s*. Contrary to the hypothesis 2, the deduction of more holistic or local positive emotional design features did not induce learners’ emotions in multimedia instruction. Interestingly, in the second measurement point, we found the significant differences for the three groups in the specific categories of positive emotions (e.g., in *strong* sub-item, *F*(2, 77) = 3.548, *p* = .034).

### Eye-tracking analysis

To analyze the eye movement data, we performed one-factorial ANOVAs with the fixation duration and fixation count on AOIs (i.e., title, textual and pictorial information, knowledge content) of the learning material as dependent factors and experimental groups as between-subject variables. Concerning the fixation of title and pictorial information, there were no significant differences among the three groups, *F*s < 1, *n.s* (see Table 2). The three groups differed significantly with regard to the fixation duration and count of textual information, *F*(2, 77) = 3.33, *p* = .041, η^2^ = .080, *F*(2, 77) = 3.67, *p* = .030, η^2^ = .087. Also, there was a significant difference in terms of the fixation duration and count of knowledge content, *F*(2, 77) = 3.44, *p* = .037, η^2^ = .082, *F*(2, 77) = 3.58, *p* = .033, η^2^ = .085. Contrast tests indicated that the group with the local interface layout of positive emotional design (LPED) spent longer fixations and more gaze counts on the textual part than the other groups, LPED-ND: Δ*M* = 29.60, *p* = .014; LPED-HPED: Δ*M* = 94.26, *p* = .041, LPED-ND: Δ*M* = 116.11, *p* = .013. Similarly, contrast tests showed that the LPED group had longer fixations and more gaze counts on the knowledge content (including the textual and pictorial information) than the other groups, LPED-ND: Δ*M* = 40.61, *p* = .014; LPED-HPED: Δ*M* = 139.02, *p* = .031, LPED-ND: Δ*M* = 154.65, *p* = .017.

**Table 2:** Means and standard deviations of eye fixation duration and eye fixation count on AOIs for the three groups

	HPED *N* = 27	LPED *N* = 27	ND *N* = 27	F	p
	*M (SD)*	*M (SD)*	*M (SD)*		
Fixation duration on title [s]	3.80 (5.99)	2.84 (2.42)	3.67 (3.32)	.34	.709
Fixation duration on texts [s]	138.70 (43.39)	159.68 (41.46)	130.08 (43.66)	3.33	.041
Fixation duration on pictures [s]	56.42 (21.99)	66.90 (24.47)	55.58 (28.11)	1.68	.193
Fixation duration on pictures and texts [s]	196.08 (59.45)	227.14 (53.52)	186.53 (62.79)	3.44	.037
Fixation count on title [s]	17.48 (24.48)	12.65 (11.71)	15.33 (16.91)	.45	.639
Fixation count on texts [s]	584.59 (171.55)	678.85 (157.17)	562.74 (166.81)	3.67	.030
Fixation count on pictures [s]	220.89 (80.06)	267.65 (104.09)	226.37 (110.71)	1.75	.180
Fixation count on pictures and texts [s]	809.63 (231.65)	948.65 (212.05)	794.00 (247.04)	3.58	.033

### Self-reports and subjective experience

To evaluate the impact of positive emotional design incorporated in the multimedia instruction on learners’ subjective feelings, we conducted one-factorial ANOVAs with the dependent variables, including invested cognitive load, perceived task difficulty, interface design score, intrinsic motivation, experienced satisfaction, and expected learning outcomes (see Table 3). With regard to invested cognitive load, it was found that there was a significant difference among the three groups, *F*(2, 77) = 6.71, *p* = .002, η^2^ = .148. Contrast tests indicated that the LPED group was significantly higher in contrast to each other group, LPED-HPED: Δ*M* = 0.72, *p* = .031, LPED-ND: Δ*M* = 1.21, *p* = .001. With regard to perceived task difficulty, there was also a significant difference among the three groups, *F*(2, 77) = 3.67, *p* = .030, η^2^ = .087. Contrast tests indicated that the interface layout of positive emotional design group (HPED and LPED) perceived higher task difficulty compared to the neutral emotional group, HPED-ND: Δ*M* = 0.80, *p* = .016, LPED-ND: Δ*M* = 0.77, *p* = .030. Additionally, with regard to the rest of the dependent variables, learners’ interface design score, internal situational motivation, experienced task satisfaction, and expected learning outcomes in the multimedia instruction designed with positive emotions (HPED and LPED) were almost slightly higher than the neutral group; so no significant differences are observed.

**Table 3:** Means and standard deviations of subjective experience for the three groups

	HPED *N* = 27	LPED *N* = 27	ND *N* = 27	F	p
	*M (SD)*	*M (SD)*	*M (SD)*		
Invested cognitive load [max = 9]	6.93 (1.21)	7.65 (0.94)	6.44 (1.42)	6.71	.002
Perceived task difficulty [max = 7]	4.30 (1.17)	4.23 (0.95)	3.56 (1.19)	3.67	.030
Interface score [max = 7]	5.07 (0.96)	4.96 (1.08)	4.81 (1.36)	.35	.707
Intrinsic motivation [max = 56]	40.85 (8.17)	42.81 (6.93)	38.96 (6.50)	1.87	.161
Experienced task satisfaction [max = 7]	4.93 (0.99)	4.85 (1.01)	4.67 (1.24)	.40	.671
Expected learning outcomes [max = 7]	3.74 (1.29)	4.08 (1.26)	3.96 (1.26)	.483	.619

## Discussion

### Benefits of the positive emotional interface design

Compared with the original multimedia design (neutral group), both groups with the positive emotion design (i.e., HPED and LPED) did not show superiority in learning outcomes; only the local interface layout of positive emotional design group (HPED) achieved significantly better performance in retention and transfer tests.

Perhaps, the specific positive emotional design might attract learners’ attention to allocate their mental effort, which is helpful for sustaining more efficient cognitive progress during learning, including selecting, organizing, elaborating, metacognitive processes, and extraneous processes (Stark et al., 2018). Therefore, learners can understand the knowledge content of multimedia material, retrieve or apply perceived information to different situations, resulting in improved problem-solving ability (Isen & Daubman, 1984; Mayer, 2005).

In line with the assumption of CATLM and emotions as learning facilitators (Moreno, 2005, 2009), the effect of interface layout of positive emotion design on learning performance is partially verified. Moreover, the result with regard to learning outcomes is consistent with the previous studies (Li et al., 2020; Münchow & Bannert, 2019; Plass et al., 2014; Um et al., 2012) that the elicitation of positive emotional design before learning or during learning maintained better scores on learners’ final test performance in multimedia instruction environment.

### No significant effect on learners’ emotional state

Regarding learners’ emotional state, the three experimental groups showed no significant emotional changes before and during the learning process. Thus, the interface layout of positive emotional design might not be an appropriate method of emotional stimulation and did not construct a direct mapping relationship with learners’ emotional state. Furthermore, the significant differences among the three groups were observed in the specific sub-items of positive emotion (e.g., strong) in the second measurement point.

The possible reason might be attributed to the positive emotional design of multimedia material. The previous studies had focused on the overall positive emotional design of multimedia program, including not only the visual appearance design, but also the redesign of knowledge content with warm colors and anthropomorphic shapes (Li et al., 2020; Mayer & Estrella, 2014; Plass et al., 2014; Stark et al., 2018; Um et al., 2012). Therefore, learners are attracted by these visual emotional elements and therefore, their positive valances are aroused. However, the main contribution of this study is to differentiate the two factors of interface design and content design and only retain the positive emotional elements into the interface layout design of material. The fact that the multimedia material for this study has fewer positive emotional parts might be explained by the impact of multi-dimensional emotional elements, which deactivates learners’ positive emotions.

The insignificant effect on learners’ emotional state in this study is contradictory to the most previous study (Mayer & Estrella, 2014; Plass et al., 2014; Stark et al., 2018; Um et al., 2012). However, some studies indicated that emotional design of learning material did not stimulate learners’ positive emotions (Heidig et al., 2015; Li et al., 2020; Park et al., 2015a, b).

### Emotional interface design and eye movements

Regarding the data analysis of eye tracking, there was a significant difference in fixation duration and fixation count of textual information and knowledge content (i.e., textual and pictorial parts) among the three groups. Further, the overall positive emotional design group (HPED) was not significantly higher than that of the neutral group. But, the local positive emotional design group (LPED) did attract more attention from learners who were willing to spend more mental efforts in multimedia learning. Moreover, the LPED group showed longer fixation duration and more gaze frequency on the textual information and knowledge content than the other groups.

On the one hand, due to the confusing effect of textual information on fixation data, learners showed significant attentional focus in not only textual information but also knowledge content. On the other hand, compared with the overall interface layout of positive emotional design, the local interface layout of positive emotional design modality might make learners experience more freedom and openness, without being restricted to allocate attentional focus to the important instructional content.

The analysis of eye tracking supports the eye-mind hypothesis that longer fixation duration and higher fixation counts on the textual part of learning material are associated with better learning outcomes in the multimedia environment (Just & Carpenter, 1976). This is consistent with the most previous studies that learners tended to process multimedia material in a text-driven manner (Hannus & Hy€on€a, 1999; Hegarty & Just, 1993; Knörzer et al., 2016a, b; Park et al., 2015a, b).

### Improved subjective experiences

Regarding invested cognitive load, the three groups differed significantly. The local interface layout of positive emotional design (LPED) group had significantly higher scores in comparison with that of the holistic positive emotional design (HPED) group and the neutral group. Thus, improved cognitive effort is facilitated by an emotional interface design. In line with the control-value theory of achievement emotions (Pekrun, 2006), academic emotion influences learning performance through the mediation of cognition and motivation. An indirect relationship between emotional design of learning material and learning outcomes seems to be found. That is, the emotional design can change and influence effort-related metacognitive experiences of learners, leading them to invest more visual attention and cognitive effort readiness to complete the task (Efklides et al., 2006; Uzun & Yıldırım, 2018). Furthermore, both the positive emotional groups presented significantly higher scores in perceived task difficulty and there was only a difference with the neutral group but not with each other. This finding contradicts the previous studies obtained by Mayer and Estrella (2014) and Plass et al. (2014). They concluded that the use of warm color and anthropomorphic design was conducive to reducing learners’ internal cognitive load. Learners had enough cognitive resources to employ advanced learning strategies to deeply process and integrate learning material, which allowed for the promotion of knowledge transfer.

Additionally, learners’ subjective experiences including interface design score, internal situational motivation, experienced task satisfaction, and expected learning outcomes are not found significant differences among the three groups. But, learners showed significant differences in specific sub-items of internal motivation (i.e., curious and interesting), which might be due to the fact that the level of activation dimension of learners’ motivation is constricted (Pekrun, 1992).

## Conclusions, implications, limitations and future work

In this study, we investigated whether the interface layout design of multimedia learning environment embedded with positive emotional elements enhanced learner’ understanding of knowledge content, changed their emotional state, facilitated their cognitive mental process, and improved their subjective learning experience. The results of the study showed that the interface layout design of learning material associated with positive emotional items did not induce students’ positive emotions in the multimedia environment. But the local interface layout of positive emotional design group invested higher cognitive effort, put more attentional focus in the relevant knowledge content module measured by eye tracking, and achieved better test performance in comparison with the overall positive emotional design group and the neutral group. Therefore, it is reasonable to believe that this design modality might be treated as a feasible visual design method in multimedia learning environment.

The most important contribution of this study was to innovatively separate the two variables of visual interface design and structured content design of multimedia learning materials. Further, we redesigned the learning material from the interface layout dimension of positive emotional design, thus improving learners’ test performance. For researchers, therefore, this study puts forward a new research question whether visual interface design or structured content design is highly associated with learning effects. The study can provide some valuable insights into the findings of the previous research, which allows for constituting a further step to promote the related researches on the positive emotional design of multimedia learning. For practitioners, they can adopt the interface layout mode of the positive emotional design and scientifically redesign the relevant stimulus elements that can trigger positive emotions according to the characteristics of learning content. As a result, learners’ internal motivation and learning outcomes are facilitated. Especially for the impact of the COVID-19 pandemic on education, practitioners might use the positive emotional interface layout to redesign the learning material, thus improving learners’ external human–computer interaction space in the distance teaching environment as much as possible. Moreover, for practitioners, three steps can be taken to redesign educational multimedia content presentation. First, the important prerequisites of designing multimedia learning materials should comply with the seven basic principles of multimedia learning, as well as get a deeper understanding of the cognitive theory of multimedia learning. Second, the local interface layout of positive emotional design modality incorporating warm colors and anthropomorphic shapes (e.g., the yellow outer frame including the anthropomorphic smiling face) should be retained. Finally, in order to maintain the visual openness and the overall beauty for instructional presentation, the selection of local frames and the proportion between frames and material content need to be appropriately adjusted.

This study has some limitations and future work can be conducted to improve these shortcomings. The participants in the study are all college students from a University in China. The focuses of multimedia learning material on scientific knowledge “the formation of lightning” and the external visual interface layout of positive emotional design paradigm are presented. Therefore, caution should be taken when the results of the study are generalized to other populations and other disciplines (Li et al., 2020; Park et al., 2015a, b). Hence, in the future, differences in participants’ socio-cultural backgrounds and individual characteristics should be considered to activate participants’ emotional valences (Knörzer et al., 2016a, b; Russell, 2003). Besides, the study is based on the external experimental environment controlled by artificial conditions and so, the positive emotional design principle of multimedia learning might be limited in the real teaching scenarios. Thus, follow-up researches should be carried out to push the findings of laboratory situation into pedagogical practice, especially in online learning environments. Additionally, the mechanism of the influence of positive emotional design on multimedia learning effects was investigated. Although eye tracking technology was utilized to provide a partial explanation for the relationship between the positive emotional design of multimedia instruction and learning outcomes, the study only used subjective scales for measuring metacognition-related emotional experience. Therefore, future research can adopt more direct and real-time multivariate mixed physiological measures (Le et al., 2018; Münchow & Bannert, 2019; Stark et al., 2018), including Electroencephalogram (EEG), Galvanic Skin Response (GSR), Heart Rate Variability (HRV), etc., to reveal the internal mechanism of positive emotional design in multimedia learning, which will subsequently be helpful in providing more comprehensive and scientific guidance for multimedia learning design.

## Data Availability

The data collected in the current study are not publicly available since they were retrieved under students’ authorization and anonymity as well as permission of relevant departments of the university.
